# CSE/H_2_S Signaling Pathways in Enhancing Muscle Function and Insulin Sensitivity During Exercise

**DOI:** 10.3390/ijms26041741

**Published:** 2025-02-18

**Authors:** Miaomiao Xu, Xiaoguang Liu, Danting Hu, Zhaowei Li, Liming Lu

**Affiliations:** 1 School of Physical Education and Health, Guangzhou University of Chinese Medicine, Guangzhou 510006, China; miaomiaoxu@gzucm.edu.cn (M.X.); 20231110459@stu.gzucm.edu.cn (D.H.); 2 South China Research Center for Acupuncture and Moxibustion, Medical College of Acu-Moxi and Rehabilitation, Guangzhou University of Chinese Medicine, Guangzhou 510006, China; 3 College of Sports and Health, Guangzhou Sport University, Guangzhou 510500, China; liuxg@gzsport.edu.cn

**Keywords:** cystathionine γ-lyase, hydrogen sulfide, muscle function, insulin sensitivity, exercise adaptation

## Abstract

Exercise plays a crucial role in maintaining metabolic health, enhancing muscle function, and improving insulin sensitivity, thereby preventing metabolic diseases such as type 2 diabetes. Emerging evidence highlights the significance of the cystathionine γ-lyase (CSE)/hydrogen sulfide (H_2_S) signaling pathway as a pivotal regulator in the molecular and physiological adaptations induced by exercise. This review comprehensively examines the biosynthesis and metabolism of H_2_S, its distribution in different muscle tissues, and the mechanisms by which CSE/H_2_S influences muscle contraction, repair, and protein synthesis. Additionally, it explores how CSE/H_2_S modulates insulin signaling pathways, glucose uptake, and lipid metabolism, thereby enhancing insulin sensitivity. The potential of H_2_S donors as exercise supplements is also discussed, highlighting their ability to improve exercise performance and metabolic health. Current research advancements, including the application of multi-omics approaches, are reviewed to provide a deeper understanding of the complex molecular networks involved. Furthermore, the challenges and future directions in CSE/H_2_S research are addressed, emphasizing the need for further mechanistic studies and clinical applications. This review underscores the therapeutic potential of targeting the CSE/H_2_S pathway to optimize the benefits of exercise and improve metabolic health.

## 1. Introduction

Exercise plays a pivotal role in maintaining and enhancing overall health, particularly concerning metabolic health, muscle function, and insulin sensitivity [1]. Regular physical activity has been shown to reduce the risk of chronic diseases such as type 2 diabetes, cardiovascular diseases, and obesity [2]. Through various mechanisms, exercise enhances metabolic flexibility, allowing the body to efficiently switch between fuel sources like carbohydrates and fats [3]. Additionally, engaging in consistent physical activity promotes muscle hypertrophy and strength, which are essential for daily functional activities and overall quality of life [4]. Furthermore, exercise improves insulin sensitivity, facilitating better glucose uptake by cells and thereby regulating blood glucose levels [5]. These multifaceted benefits underscore the critical importance of incorporating exercise into daily routines for long-term health maintenance and disease prevention.

The beneficial effects of exercise are underpinned by a complex array of molecular and physiological adaptations [6]. Understanding these adaptations requires an exploration of the intricate signaling pathways that are activated in response to physical activity [7,8,9,10]. For instance, exercise induces the activation of AMP-activated protein kinase (AMPK), which plays a key role in energy homeostasis by enhancing glucose uptake and fatty acid oxidation [10]. Similarly, the mitogen-activated protein kinase (MAPK) pathways are involved in regulating muscle growth and repair [9]. These signaling cascades not only facilitate immediate responses to exercise but also drive long-term adaptations such as increased mitochondrial biogenesis and improved oxidative capacity [7,8]. By elucidating these underlying mechanisms, we can better comprehend how exercise confers its health benefits and potentially identify novel therapeutic targets for metabolic and muscular disorders.

Cystathionine γ-lyase (CSE) is a critical enzyme in the biosynthesis of hydrogen sulfide (H_2_S), a gaseous signaling molecule [11]. CSE catalyzes the conversion of cystathionine to cysteine, producing H_2_S as a byproduct [12]. This enzyme is predominantly expressed in the cardiovascular system, liver, and skeletal muscles, highlighting its significant role in various physiological processes [13,14,15]. Beyond its enzymatic activity, CSE is implicated in regulating cellular redox status and modulating inflammatory responses [16,17]. The regulation of CSE expression and activity is influenced by several factors, including oxidative stress and hormonal signals, which in turn affect H_2_S production [18]. Understanding the regulation of CSE is essential for comprehending how H_2_S levels are maintained and modulated in different tissues.

H_2_S has emerged as a vital gasotransmitter, alongside nitric oxide (NO) and carbon monoxide (CO), playing diverse roles in cellular signaling [19]. H_2_S is involved in numerous physiological processes, including vasodilation, neurotransmission, and cytoprotection [20]. In the context of exercise, H_2_S has been shown to enhance muscle blood flow, thereby improving oxygen and nutrient delivery to active tissues [21]. Additionally, H_2_S modulates mitochondrial function, promoting efficient energy production and reducing oxidative stress [22]. Its anti-inflammatory properties contribute to the attenuation of exercise-induced muscle damage and facilitate recovery [23]. Moreover, H_2_S interacts with insulin signaling pathways, enhancing insulin sensitivity and glucose uptake in skeletal muscles [11,24]. The multifaceted roles of H_2_S underscore its significance in mediating the beneficial effects of exercise on muscle function and metabolic health.

This narrative review aims to clarify the role of CSE/H_2_S signaling in enhancing muscle function and insulin sensitivity during exercise. By synthesizing current evidence, we will examine how H_2_S mediates adaptive responses to physical activity through its influence on key molecular pathways, interactions with gasotransmitters, and regulation of processes such as mitochondrial biogenesis, antioxidant defense, and inflammation. Additionally, we will explore the therapeutic potential of targeting the CSE/H_2_S axis to maximize exercise benefits and address metabolic and muscular disorders.

## 2. Methods

To ensure transparency and replicability, a comprehensive literature search was conducted across the PubMed, Scopus, and Web of Science databases, focusing on publications from 2015 to 2025 and limited to English-language articles. The search involved a combination of keywords, including “Cystathionine γ-Lyase”, “Hydrogen Sulfide”, “Muscle Function”, “Insulin Sensitivity”, and “Exercise Adaptation”, optimized with Boolean operators. In addition to the database searches, relevant articles and reference lists from existing reviews were manually checked to enhance the thoroughness of the review.

The inclusion criteria were broadened to studies examining the role of cystathionine γ-lyase and hydrogen sulfide in muscle function, insulin sensitivity, and exercise adaptation, including both human and animal studies, while non-peer-reviewed articles, conference abstracts, and theoretical papers were excluded. A two-stage screening process was employed to assess study eligibility: an initial screening of titles and abstracts followed by a full-text review conducted by two independent reviewers. After completing this rigorous selection process, 159 relevant articles were included in this narrative review.

## 3. Biological Basis of CSE/H_2_S Signaling

### 3.1. Synthesis and Metabolism of H_2_S

**Figure 1:**
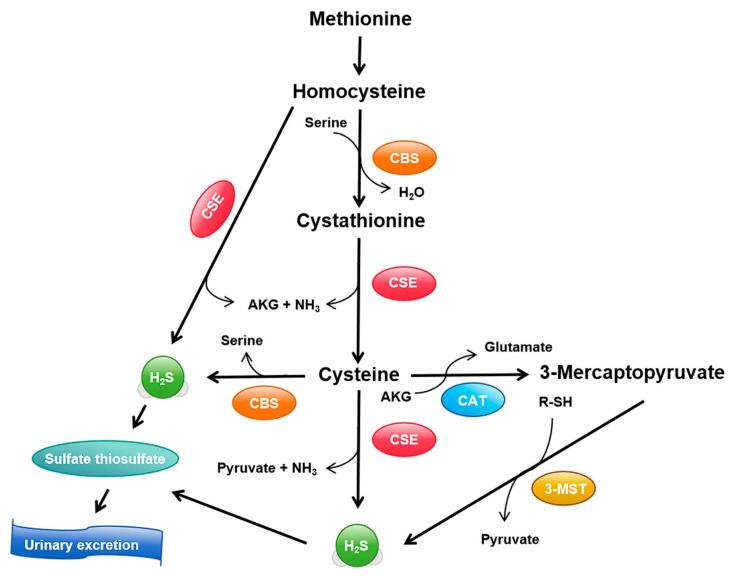
H_2_S biosynthesis and metabolic pathways. This diagram illustrates the key enzymes and metabolic steps involved in H_2_S production. Methionine is converted to homocysteine, which is then catalyzed by CBS to form cystathionine. CSE further converts cystathionine to cysteine, producing H_2_S. Cysteine can also be converted into 3-mercaptopyruvate by CAT, which is then acted upon by 3-MST. H_2_S contributes to the formation of sulfate and thiosulfate, which are ultimately excreted in the urine. CBS, cystathionine β-synthase; CSE, cystathionine γ-lyase; CAT, cysteine aminotransferase; 3-MST, 3-mercaptopyruvate sulfurtransferase; H_2_S, hydrogen sulfide; R-SH, sulfur-hydryl group; AKG, α-ketoglutarate.

H_2_S production primarily relies on three key enzymes: cystathionine γ-lyase (CSE), cystathionine β-synthase (CBS), and 3-mercaptopyruvate sulfurtransferase (3-MST) [25]. These enzymes collaborate to produce H_2_S from various metabolic precursors. CSE catalyzes the conversion of cystathionine to cysteine and H_2_S in the CSE/CBS pathway, serving as one of the main routes for H_2_S generation [26]. CBS, predominantly expressed in the central nervous system, generates H_2_S from serine and cysteine [27]. 3-MST operates in the cytoplasm and mitochondria, contributing to H_2_S production via reactions with 3-mercaptopyruvate [28] (Figure 1). The synergistic action of these three enzymes ensures proper generation of H_2_S in various tissues and cell types, including skeletal muscles [29]. Additionally, non-enzymatic pathways, such as thiosulfate reduction, contribute to small amounts of H_2_S production [30].

Exercise has been shown to influence the activity of these enzymes, affecting H_2_S synthesis. Acute exercise increases CSE expression in skeletal muscles, promoting H_2_S production in response to the immediate metabolic demands of physical activity [31]. In contrast, chronic exercise induces long-term adaptations by upregulating CSE activity, enhancing the muscle’s ability to generate H_2_S during sustained or repeated exercise bouts [24]. This modulation is essential for supporting muscle function and improving metabolic efficiency during exercise.

H_2_S synthesis and turnover are tightly regulated by substrate availability, oxidative stress, and hormonal signals. For example, insulin and glucagon modulate H_2_S synthesis by influencing CSE and CBS activity, with insulin typically promoting H_2_S production in skeletal muscle during exercise [32,33]. Oxidative stress, a common result of intense or prolonged exercise, also affects the regulation of H_2_S synthesis, with high levels of reactive oxygen species (ROS) potentially enhancing CSE expression in response to cellular damage [34,35]. The homeostasis of H_2_S is critical, as excessive accumulation could lead to toxic effects, including mitochondrial dysfunction and impaired cellular signaling [36]. While H_2_S exerts beneficial effects at physiological levels, its accumulation at supra-physiological levels may interfere with normal cellular processes, leading to potential toxicity [37]. Therefore, feedback mechanisms are essential to control the rate of H_2_S synthesis and degradation, preventing detrimental effects on muscle cells and overall metabolic health.

The degradation of H_2_S primarily occurs through reactions with biological molecules, such as binding to metal ions and proteins or mitochondrial oxidation [38]. These processes ensure that H_2_S levels remain within a physiologically optimal range, supporting its role in exercise adaptations without causing toxicity.

### 3.2. Distribution and Expression in Muscle Tissues

The expression and functional roles of CSE, CBS, and 3-MST vary across different muscle tissues, influencing muscle function and metabolism. In skeletal muscle, CSE expression is particularly high in fast-twitch fibers, which is crucial for regulating muscle contraction and energy metabolism during high-intensity activities [39]. CBS, on the other hand, is predominantly expressed in cardiac muscle, where it plays a vital role in regulating blood flow and protecting myocardial cells during cardiac stress [40]. 3-MST is found in both skeletal and cardiac muscles, but its precise mechanisms of action remain less understood and require further exploration [41].

Exercise significantly modulates the expression of these enzymes, adapting them to different physiological demands. Endurance training typically increases CSE expression in skeletal muscle, enhancing H_2_S production, which improves blood flow and oxygen delivery to active tissues [42]. High-intensity interval training (HIIT), known for its metabolic demands, has been shown to activate CBS and 3-MST, boosting H_2_S production to support cellular energy demands during intense bouts of exercise [43]. Furthermore, long-term regular exercise can lead to upregulation of CSE gene expression, thereby enhancing the capacity for H_2_S synthesis and promoting muscle adaptation to sustained physical activity [24].

Different types of exercise, including resistance training, may also influence the expression of these enzymes, though the specific mechanisms and outcomes vary between exercise modalities. For instance, resistance training might alter the expression of CSE and CBS differently than endurance exercise due to the distinct metabolic and mechanical stresses involved [44]. Furthermore, it is essential to note that much of the research on these signaling pathways has been conducted in rodent models, and species differences should be considered when translating these findings to human physiology.

### 3.3. H_2_S as a Signaling Molecule

H_2_S exerts its effects on cellular targets through various mechanisms, including post-translational modifications such as sulfhydration, and interactions with other signaling molecules like NO and ROS [45,46,47]. Sulfhydration, the process by which H_2_S forms sulfide bonds with protein cysteine residues, alters protein function and activity, thus modulating cellular signaling pathways [45,46,47]. This modification plays a critical role in regulating several physiological processes, including mitochondrial function and stress responses during exercise.

In terms of vasodilation, H_2_S enhances the effects of NO by regulating its synthesis and bioavailability, contributing to increased blood flow during physical activity, which improves oxygen and nutrient delivery to muscle tissues [21]. H_2_S also acts as a potent scavenger of ROS, helping to mitigate oxidative stress and prevent cellular damage induced by exercise [48]. This antioxidant role is crucial for maintaining cellular integrity during intense physical exertion, supporting recovery and minimizing muscle damage.

H_2_S influences several key cellular pathways involved in exercise physiology, including antioxidant defenses, anti-inflammatory responses, and metabolic regulation. By activating the nuclear factor erythroid 2-related factor 2 (Nrf2) signaling pathway, H_2_S promotes the expression of antioxidant enzymes such as glutathione peroxidase (GPx) and superoxide dismutase (SOD), thereby reducing oxidative stress during and after exercise [21]. Furthermore, H_2_S plays a significant role in regulating inflammation by inhibiting the production of pro-inflammatory cytokines like TNF-α and IL-6, which are elevated during intense exercise and contribute to muscle soreness and injury [49].

Metabolically, H_2_S modulates cellular energy homeostasis by activating the AMPK and peroxisome proliferator-activated receptor gamma coactivator 1-alpha (PGC-1α) signaling pathways, promoting mitochondrial biogenesis and enhancing fatty acid oxidation [48]. These adaptations are vital for improving endurance performance and muscle recovery. Additionally, H_2_S enhances insulin signaling, improving glucose uptake and utilization and ultimately increasing insulin sensitivity in skeletal muscle [50,51].

While the beneficial roles of H_2_S in exercise physiology are clear, it is also important to acknowledge the potential negative effects of excessive H_2_S signaling. High levels of H_2_S may interfere with ROS signaling, disrupt apoptosis regulation, and lead to cellular dysfunction [52]. Thus, it is crucial to maintain a balanced level of H_2_S to prevent negative outcomes. The modulation of H_2_S levels during exercise, as well as its complex interaction with other signaling molecules like NO and ROS, underscores the need for further research to better understand the full scope of its physiological effects.

## 4. Exercise-Induced Molecular and Physiological Adaptations

### 4.1. Enhancements in Muscle Function

Exercise promotes increases in muscle strength and endurance through a variety of molecular mechanisms. Strength training stimulates muscle protein synthesis and fiber hypertrophy by activating the mammalian target of rapamycin (mTOR) pathway in response to mechanical tension, metabolic stress, and muscle damage [50]. Additionally, exercise-induced mechanical stress activates satellite cells, which facilitate muscle repair and regeneration, thereby enhancing muscle strength and volume [53]. In contrast, endurance training activates the AMPK signaling pathway, which increases mitochondrial biogenesis and fatty acid oxidation capacity, leading to enhanced muscle endurance [54]. PGC-1α, a key regulator of mitochondrial biogenesis, upregulates the expression of genes linked to oxidative metabolism, thus improving the muscle’s aerobic capacity [55].

The effects of exercise vary depending on the type, intensity, and duration of the activity. Endurance training leads to a shift in muscle fiber types from glycolytic to oxidative fibers, which is driven by changes in mitochondrial density and enzyme activity within the muscle fibers [7]. Slow-twitch fibers (Type I) have higher mitochondrial content and greater oxidative enzyme activity, allowing for more efficient oxygen utilization and enhanced endurance performance [56]. Exercise-induced signaling molecules, such as Ca^2+^/calmodulin-dependent kinase (CaMK) and myocyte enhancer factor 2 (MEF2), regulate the conversion of muscle fibers to types with greater endurance characteristics, improving overall muscle function and performance [7,57,58].

### 4.2. Improvements in Insulin Sensitivity

Insulin plays a critical role in glucose uptake by facilitating the translocation of GLUT4 to the muscle cell membrane, increasing intracellular glucose uptake [59]. Exercise enhances insulin sensitivity through two main pathways: first, by increasing muscle cells’ responsiveness to insulin, and second, by promoting GLUT4 translocation via insulin-independent pathways [60]. The activation of AMPK during exercise is key to GLUT4 translocation, which improves glucose uptake and helps regulate blood glucose levels even in conditions where insulin signaling is impaired [61].

Exercise influences multiple signaling pathways involved in insulin sensitivity. Exercise-induced activation of the PI3K/Akt pathway further enhances insulin sensitivity by promoting glucose uptake and metabolism [62]. Additionally, the AMPK and PGC-1α pathways promote mitochondrial biogenesis and fatty acid oxidation, improving energy metabolism efficiency and enhancing insulin sensitivity [63]. Exercise also lowers systemic inflammation by reducing inflammatory cytokine production from adipose tissue, further promoting insulin action [64]. These effects are influenced by exercise intensity, as higher-intensity exercise may provide greater improvements in insulin sensitivity by activating more robust metabolic and inflammatory responses.

### 4.3. Multi-Omics Approaches in Exercise Research

Multi-omics technologies, which integrate genomic, transcriptomic, proteomic, and metabolomic data, have made significant contributions to understanding exercise-induced adaptations. These approaches have revealed the complex, multi-layered molecular networks underlying exercise responses, highlighting the role of H_2_S and other signaling molecules in these processes. For instance, genomics, through genome-wide association studies (GWAS), has revealed genetic variations associated with individual exercise capacity [65], while transcriptomics provides insights into gene expression changes before and after exercise [66,67]. Proteomics, using mass spectrometry, uncovers dynamic changes in protein expression, post-translational modifications, and protein–protein interactions in response to exercise [68,69]. Metabolomics offers a comprehensive view of how exercise affects cellular metabolism, identifying changes in metabolites related to energy metabolism, oxidative stress, and inflammation [70,71,72] (Table 1).

In the context of CSE/H_2_S signaling, multi-omics data offer insights into how H_2_S regulates key pathways that mediate muscle function and insulin sensitivity during exercise. For example, multi-omics studies have shown that endurance training upregulates genes and proteins related to mitochondrial function and fatty acid metabolism, processes heavily influenced by H_2_S [73,74]. Metabolomic studies have also demonstrated that H_2_S supplementation enhances both energy metabolism and antioxidant capacity, contributing to better endurance and muscle recovery [75]. These findings underscore the critical role of multi-omics approaches in advancing our understanding of how H_2_S modulates exercise adaptations at the molecular level.

By integrating these multi-omics insights with the study of CSE/H_2_S signaling, we can better understand how exercise-induced molecular networks are regulated. The use of multi-omics technologies also allows us to identify biomarkers of H_2_S activity, which could serve as useful tools in assessing an individual’s response to exercise and tailoring personalized exercise regimens. As the field progresses, multi-omics methods will continue to refine our understanding of the molecular mechanisms that drive exercise adaptations and improve health outcomes.

**Table 1:** Application of multi-omics approaches in exercise and CSE/H_2_S research.

Omics Technology	Application in Exercise Research	Key Findings and Insights	References
Genomics	Genome-wide association studies (GWAS) to identify genetic variations related to exercise capacity	Detected genetic variations that influence exercise performance and adaptive responses, helping predict individual exercise capacity	[76,77]
Transcriptomics	RNA sequencing to analyze gene expression changes before and after exercise	Revealed key genes regulating muscle adaptation, mitochondrial function, and metabolism	[66,67,78]
Proteomics	Mass spectrometry to identify protein expression and post-translational modifications	Identified proteins involved in muscle repair, protein synthesis, and mitochondrial biogenesis, highlighting adaptive responses to exercise	[68,69,78]
Metabolomics	High-throughput metabolite analysis to study exercise’s effects on metabolism	Uncovered metabolite changes related to energy metabolism, oxidative stress, and inflammation, highlighting metabolic adaptation	[70,71,72,75]
Integrated Multi-Omics	Combining genomics, transcriptomics, proteomics, and metabolomics to study exercise adaptations	Integrated data revealed key insights into mitochondrial function, fatty acid metabolism, and antioxidant pathways	[79,80]

## 5. Mechanisms of CSE/H_2_S in Enhancing Muscle Function

### 5.1. Regulation of Muscle Contraction and Relaxation

**Figure 2:**
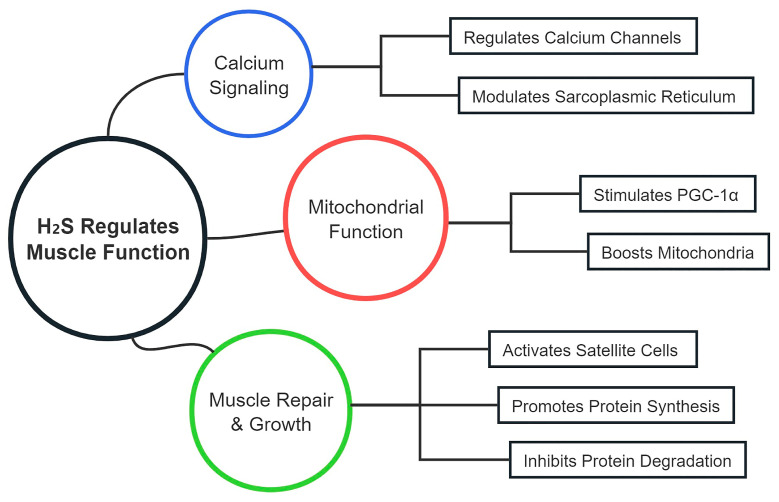
Mechanisms of H_2_S in regulating muscle function. This figure illustrates how H_2_S regulates muscle function by modulating calcium signaling, mitochondrial biogenesis, and protein synthesis/degradation. H_2_S enhances muscle contraction and relaxation by influencing calcium channels and the sarcoplasmic reticulum. Additionally, H_2_S promotes mitochondrial function by stimulating key regulators of mitochondrial biogenesis and reducing oxidative stress. H_2_S also plays a role in muscle repair and regeneration through activation of satellite cells and modulation of the microenvironment, and it regulates muscle growth by influencing protein synthesis pathways and inhibiting protein degradation.

Calcium ions (Ca^2+^) are crucial for muscle contraction and relaxation by regulating the interaction between actin and myosin filaments [81]. H_2_S modulates calcium signaling within muscle cells by altering the activity of calcium channels and transporters. For instance, H_2_S can enhance L-type calcium channel activity, increasing calcium influx during muscle contraction [82,83] (Figure 2, Table 2). However, the role of H_2_S in calcium signaling needs further investigation, particularly in different muscle fiber types (e.g., fast-twitch vs. slow-twitch fibers), as their calcium handling mechanisms may vary, affecting H_2_S’s impact on contraction and relaxation. Moreover, species differences in calcium handling (rodents vs. humans) should be addressed, as much of the current research is based on animal models.

Additionally, H_2_S modulates the function of the sarcoplasmic reticulum (SR), regulating ryanodine receptors (RyR) and sarcoplasmic/endoplasmic reticulum calcium ATPases (SERCA) to facilitate efficient calcium release and reuptake during muscle contractions [84]. This regulation ensures that muscle fibers can contract and relax efficiently, improving muscle endurance and performance [85,86]. However, a comparison of H_2_S’s effects with other established regulators, such as NO or AMPK, in calcium signaling could help clarify its relative importance in muscle function.

Mitochondria are critical for ATP production through oxidative phosphorylation [87], and H_2_S has been implicated in enhancing mitochondrial biogenesis and efficiency. Research comparing H_2_S with PGC-1α and AMPK (two key regulators of mitochondrial function) is necessary to avoid overgeneralization, as these pathways are well-established in mitochondrial biogenesis [52,88]. H_2_S stimulates key transcription factors like PGC-1α and NRF1, promoting mitochondrial formation and improving muscle energy capacity [89] (Figure 2, Table 2). While H_2_S enhances mitochondrial function, a more direct comparison with these well-characterized regulators could strengthen the evidence supporting H_2_S’s role in mitochondrial efficiency.

Furthermore, H_2_S also reduces ROS production and acts as an electron donor in the electron transport chain (ETC), thereby boosting ATP synthesis while minimizing oxidative damage [89,90]. The comparison with other mitochondrial regulators like AMPK, which also modulates mitochondrial function under exercise conditions, is important to understand whether H_2_S’s role is distinct or complementary.

### 5.2. Muscle Repair and Regeneration

Satellite cells play a vital role in muscle repair and regeneration post-exercise [92,93] (Figure 2, Table 2). H_2_S activates satellite cells by modulating key signaling pathways such as Notch, Wnt, and IGF-1/Akt, promoting their proliferation and differentiation into myoblasts [23,94]. This activation is crucial for muscle regeneration following strenuous exercise. However, the effects of H_2_S on different muscle fiber types during regeneration should be further explored, as satellite cells in different fiber types (e.g., fast-twitch vs. slow-twitch) might respond differently to H_2_S signaling, influencing the repair process [23].

Additionally, H_2_S influences the extracellular matrix and inflammatory cytokines, modulating the tissue repair environment and enhancing satellite cell activation [89,96]. The regulation of matrix metalloproteinases (MMPs) and TGF-β by H_2_S is vital for tissue remodeling [17,96]. Nevertheless, a more detailed comparison of H_2_S’s effects on muscle repair with other signaling molecules like NO, which also plays a role in muscle regeneration [23], would provide a broader understanding of H_2_S’s role in muscle repair.

Apoptosis is a significant factor in muscle integrity post-exercise, and H_2_S exhibits anti-apoptotic effects by inhibiting pro-apoptotic signaling pathways such as the mitochondrial and death receptor pathways [102,103]. This protection of muscle integrity during recovery is essential for maintaining muscle function and preventing muscle wasting. On the other hand, the dose-dependent effects of H_2_S on apoptosis [119], particularly in response to different exercise intensities, require further investigation to assess whether higher doses of H_2_S can inhibit excessive apoptosis during high-intensity training.

### 5.3. Protein Synthesis and Degradation

Anabolic signaling pathways, particularly mTOR, are central to muscle growth and hypertrophy. H_2_S influences mTOR signaling to promote muscle protein synthesis by enhancing the translation of mRNA into proteins [23,108]. However, exercise intensity-dependent effects on mTOR signaling should be incorporated, as different exercise modalities (e.g., endurance vs. resistance training) can have varying impacts on mTOR activation and subsequent protein synthesis [110]. The role of H_2_S in modulating these exercise-dependent variations in mTOR signaling should be better elucidated.

H_2_S also affects myogenic regulatory factors (MRFs) such as MyoD and myogenin, which are crucial for muscle differentiation and growth [101]. However, the impact of H_2_S on MRFs in different fiber types under various exercise conditions should be clarified, as fast-twitch and slow-twitch fibers may have different responses to H_2_S in terms of growth and hypertrophy.

In addition to promoting anabolic processes, H_2_S inhibits the catabolic pathways responsible for muscle protein degradation. H_2_S modulates the ubiquitin–proteasome system (UPS) and autophagy–lysosome pathway (ALP), reducing muscle protein breakdown during and after exercise [115]. Specifically, H_2_S suppresses the expression of E3 ubiquitin ligases, such as MuRF1 and MAFbx/atrogin-1, key components in the UPS, thereby preserving muscle mass and function [117]. The dose-dependent effects of H_2_S on protein degradation and its potential interactions with other metabolic regulators like AMPK and PGC-1α should be explored to gain a comprehensive understanding of how H_2_S modulates both anabolic and catabolic processes.

Furthermore, H_2_S interacts with FoxO transcription factors, which are involved in promoting catabolic processes. By inhibiting FoxO activity, H_2_S reduces the expression of genes involved in protein degradation, thereby maintaining muscle integrity and preventing atrophy [114]. Further investigation is needed to determine how H_2_S’s dual regulation of anabolic and catabolic pathways balances muscle growth and prevents muscle loss, particularly in response to different exercise intensities.

## 6. Mechanisms of CSE/H_2_S Signaling in Enhancing Insulin Sensitivity During Exercise

### 6.1. Regulation of Insulin Signaling

**Figure 3:**
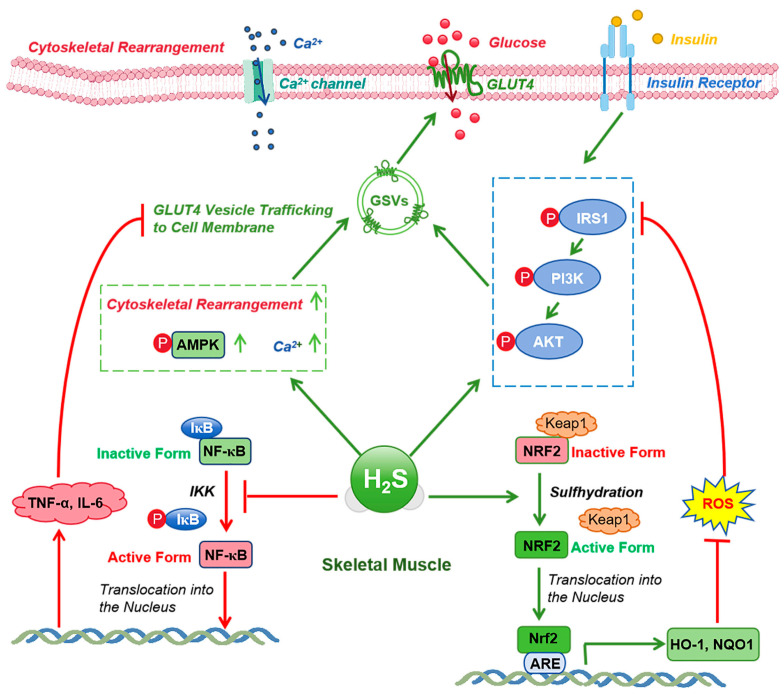
Mechanisms of H_2_S in enhancing insulin sensitivity in skeletal muscle. This figure illustrates the molecular mechanisms by which H_2_S enhances insulin sensitivity in skeletal muscle. H_2_S activates the AMPK and PI3K/AKT signaling pathways, facilitating GLUT4 translocation to the cell membrane and enhancing glucose uptake. It also promotes cell membrane remodeling, calcium ion influx, and further GLUT4 insertion into the membrane. Additionally, H_2_S exerts anti-inflammatory and antioxidant effects by activating NRF2 and inhibiting NF-κB, thereby reducing inflammatory cytokines and improving insulin signaling. The inhibitor IKK phosphorylates IκB, leading to the release and nuclear translocation of NF-κB. KEAP1 regulates NRF2 activity, and antioxidant effects are further supported by the activation of ARE and the expression of HO-1 and NQO1. AMPK, AMP-activated protein kinase; PI3K, phosphoinositide 3-kinase; AKT, protein kinase B; GLUT4, glucose transporter type 4; NRF2, nuclear factor erythroid 2-related factor 2; NF-κB, nuclear factor kappa B; IKK, IκB kinase; IκB, IκB protein; KEAP1, Kelch-like ECH-associated protein 1; ARE, antioxidant response element; HO-1, heme oxygenase 1; NQO1, NAD(P)H quinone dehydrogenase 1.

Insulin plays a vital role in regulating glucose homeostasis through the insulin receptor (IR) and downstream pathways like the PI3K/Akt cascade. H_2_S has been shown to modulate insulin sensitivity by influencing IR autophosphorylation and the activity of downstream signaling molecules such as IRS-1 and PI3K/Akt, which enhance glucose uptake and metabolism in muscle tissues [120,121,122,123] (Figure 3). However, H_2_S’s effects on insulin sensitivity extend beyond muscle tissue; it has also been observed to influence insulin signaling in adipose and hepatic tissues. For instance, in adipose tissue, H_2_S can reduce the expression of phosphatase and tensin homolog (PTEN), a negative regulator of the PI3K pathway, thus facilitating insulin signaling and improving systemic insulin sensitivity [124,125]. Additionally, H_2_S’s effect on GLUT4 translocation has been primarily studied in muscle tissues, but evidence suggests that it may also enhance GLUT4 translocation in adipocytes, supporting insulin action in both muscle and fat tissues [121,126]. This dual effect on different tissues underscores the complexity of H_2_S’s role in regulating insulin sensitivity. However, the mechanisms underlying this modulation in various tissues are not fully understood and warrant further investigation.

### 6.2. Regulation of Glucose and Lipid Metabolism

H_2_S regulates key metabolic pathways involved in glucose and lipid metabolism, providing crucial insights into its role in metabolic flexibility. One of its primary actions is enhancing glycolysis and gluconeogenesis, two opposing metabolic processes central to glucose homeostasis. H_2_S upregulates enzymes involved in glycolysis, such as hexokinase (HK) and phosphofructokinase-1 (PFK-1), to accelerate glucose utilization during exercise [127,128]. In contrast, in the liver, H_2_S enhances gluconeogenesis by promoting enzymes like glucose-6-phosphatase (G6Pase) and pyruvate carboxylase (PC) [127]. While the role of H_2_S in glycolysis and gluconeogenesis is becoming clearer, its effect on other regulators, such as the transcription factor PPARs, which are central to lipid metabolism, needs further exploration to understand its impact on fatty acid oxidation and adipose tissue accumulation [129].

Moreover, H_2_S has been shown to influence fatty acid oxidation by enhancing the activity of key enzymes involved in mitochondrial β-oxidation [130]. This action could contribute to improved insulin sensitivity by reducing excessive lipid accumulation in muscle and adipose tissues, a key factor in the development of insulin resistance. However, when compared to other regulators of fatty acid metabolism such as AMPK and PGC-1α, the unique contribution of H_2_S remains underexplored and warrants deeper scrutiny, especially in the context of varying exercise intensities and durations [129,131].

### 6.3. Anti-Inflammatory and Antioxidant Effects

Chronic low-grade inflammation, often driven by pro-inflammatory cytokines such as TNF-α and IL-6, is a major contributor to insulin resistance. H_2_S can reduce systemic inflammation by modulating the NF-κB signaling pathway, which regulates the production of these cytokines. Studies have shown that H_2_S inhibits the NF-κB pathway, which subsequently lowers inflammation and improves insulin sensitivity in various tissues, including muscle, liver, and adipose tissue [132,133]. Additionally, H_2_S activates the Nrf2 signaling pathway, which is involved in regulating the expression of antioxidant genes. This results in enhanced antioxidant enzyme activity, which counteracts oxidative stress that could otherwise impair insulin signaling [134,135] (Figure 3). Importantly, the dual anti-inflammatory and antioxidant effects of H_2_S support its potential therapeutic role in preventing metabolic diseases.

However, while the molecular mechanisms underlying H_2_S’s anti-inflammatory and antioxidant effects are becoming clearer, there is a need for more detailed investigations into dose-dependent responses. For instance, high doses of H_2_S may have unintended metabolic effects, such as altering ROS production in a way that could interfere with cellular redox balance or inflammation regulation. More research is needed to understand how different dosages of H_2_S influence both inflammation and oxidative stress, as well as how these effects vary between tissues. Furthermore, there is a need to explore how H_2_S interacts with other signaling molecules, such as NO and ROS, in the regulation of inflammation and insulin sensitivity [136]. The interplay between H_2_S and these other mediators could offer new avenues for targeted interventions to improve metabolic health and prevent insulin resistance.

In summary, while H_2_S’s role in enhancing insulin sensitivity and improving metabolic health during exercise is supported by increasing evidence, many aspects of its mechanisms remain unclear, particularly regarding its tissue-specific effects and dose-dependent actions. Future studies should aim to clarify how H_2_S interacts with other signaling pathways and assess the long-term effects of supplementation to better understand its full therapeutic potential.

## 7. H_2_S Supplementation in Exercise

### 7.1. Types of H_2_S Donors

Synthetic donors are widely used in experimental and clinical studies to provide controlled release of H_2_S. Common synthetic H_2_S donors include sodium hydrosulfide (NaHS) and GYY4137. NaHS is a fast-releasing donor that rapidly elevates in vivo H_2_S concentrations, making it suitable for acute studies [137]. On the other hand, GYY4137 is a slow-releasing donor, with kinetics closer to physiological conditions, which makes it ideal for long-term studies and clinical applications [138]. Other synthetic donors, such as AP39 and JK1, release H_2_S through different chemical mechanisms, offering diverse tools for research purposes [139] (Table 3).

### 7.2. Effects on Exercise Performance and Metabolic Health

**Figure 4:**
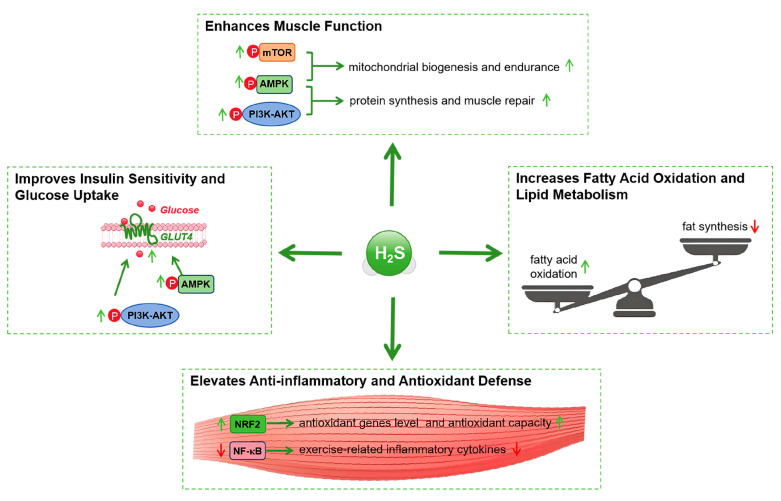
Impact of H_2_S supplementation on exercise-induced adaptations. This figure illustrates how H_2_S supplementation enhances exercise adaptations. H_2_S improves muscle function by promoting muscle contraction, repair, mitochondrial biogenesis, and protein synthesis while inhibiting protein degradation through the AMPK, mTOR, and PI3K/AKT pathways. It also enhances insulin sensitivity by facilitating GLUT4 translocation and glucose uptake. H_2_S improves metabolic health by promoting fatty acid oxidation, reducing fat synthesis, and modulating lipid metabolism. Additionally, H_2_S exerts anti-inflammatory and antioxidant effects by activating NRF2 and inhibiting NF-κB, reducing inflammation and oxidative stress, thereby enhancing insulin signaling and overall metabolic health. AMPK, AMP-activated protein kinase; mTOR, mechanistic target of rapamycin; PI3K, phosphoinositide 3-kinase; AKT, protein kinase B; GLUT4, glucose transporter type 4; NRF2, nuclear factor erythroid 2-related factor 2; NF-κB, nuclear factor kappa B.

Numerous studies have evaluated the effects of H_2_S supplementation on endurance, strength, and recovery during exercise. H_2_S donors, such as GYY4137, have been shown to significantly enhance oxygen consumption and running time during endurance exercises, suggesting positive effects on aerobic capacity [24,116,144]. Additionally, supplementation has demonstrated the ability to increase muscle strength and explosive power, promoting muscle fiber hypertrophy [24]. Furthermore, H_2_S supplementation has facilitated faster recovery from exercise by reducing inflammation and oxidative stress, thereby decreasing muscle soreness and damage [145] (Figure 4).

H_2_S donors have also been observed to improve insulin sensitivity, glucose metabolism, and lipid profiles. Studies indicate that H_2_S supplementation enhances insulin sensitivity, promoting glucose uptake and utilization, thereby lowering blood glucose levels [129]. In lipid metabolism, H_2_S donors have been shown to improve lipid profiles by stimulating fatty acid oxidation and inhibiting fat synthesis, which helps reduce adipose tissue accumulation [140] (Figure 4). These metabolic improvements may help prevent metabolic syndrome and type 2 diabetes, thereby promoting overall metabolic health.

However, it is important to critically assess the potential risks associated with H_2_S supplementation. One significant consideration is the dose-dependent toxicity of H_2_S, which can vary depending on the dose and duration of supplementation [37]. While acute supplementation has shown benefits, prolonged or excessive H_2_S exposure may lead to unintended metabolic effects, such as interference with other cellular signaling pathways [146]. Additionally, interactions between H_2_S and other gasotransmitters, such as NO or CO, warrant further investigation, as these molecules may modulate each other’s effects [21], potentially altering their efficacy or contributing to side effects.

### 7.3. Mechanistic Insights from Supplementation Studies

H_2_S supplementation exerts its effects during exercise through multiple molecular pathways. Firstly, H_2_S activates the AMPK and PI3K/Akt signaling pathways, facilitating GLUT4 translocation and glucose uptake, thereby improving insulin sensitivity [32,121]. Additionally, H_2_S activates the mTOR pathway, promoting protein synthesis and muscle growth [39]. In terms of anti-inflammatory and antioxidant effects, H_2_S activates and inhibits the Nrf2 and NF-κB signaling pathways, respectively, reducing inflammation and oxidative stress, thus protecting insulin signaling molecules [147].

In terms of exercise adaptations, H_2_S supplementation has been shown to enhance mitochondrial biogenesis and fatty acid oxidation, which complements the effects of endurance training [148]. During strength training, H_2_S works synergistically with exercise to promote muscle protein synthesis and muscle fiber hypertrophy, leading to increased muscle strength and mass [107]. This combined effect of exercise and H_2_S supplementation not only improves performance but also optimizes metabolic adaptations, contributing to better overall health.

Despite the promising results observed with H_2_S supplementation, it is important to evaluate whether these benefits are physiologically relevant, particularly when supra-physiological doses are used in animal models. The pharmacokinetics and bioavailability of synthetic H_2_S donors should be compared to endogenous production levels of H_2_S during exercise, which could provide insights into the real-world relevance of supplementation. Furthermore, dose–response relationships and the long-term safety of H_2_S supplementation must be better understood to determine optimal dosing strategies that maximize benefits while minimizing potential risks [149].

In conclusion, while H_2_S supplementation holds considerable potential for enhancing exercise performance and improving metabolic health, further research is needed to evaluate its long-term effects, optimal dosages, and potential interactions with other signaling pathways. The balance between efficacy and safety must be carefully addressed [150] in order to provide a realistic and well-rounded assessment of H_2_S supplementation in exercise physiology.

## 8. Current Research, Technological Advances, and Future Directions

### 8.1. Summary of Current Research

In recent years, numerous significant discoveries have been made regarding the role of CSE/H_2_S in enhancing muscle function and insulin sensitivity during exercise [24,151]. Studies have shown that CSE generates H_2_S, thereby regulating energy metabolism and signaling pathways within muscle cells, which promotes the enhancement of muscle endurance and strength [152]. Additionally, H_2_S activates the PI3K/Akt and AMPK signaling pathways, increasing insulin receptor sensitivity, facilitating GLUT4 translocation, and enhancing glucose uptake efficiency [32,121]. Further research has revealed that H_2_S possesses anti-inflammatory and antioxidant properties, reducing exercise-induced oxidative stress and inflammatory responses, thereby protecting the function of insulin signaling molecules [84,153].

Despite existing studies elucidating the multiple mechanisms by which CSE/H_2_S operates during exercise, several knowledge gaps and controversies remain. Firstly, the specific mechanisms of H_2_S action in different types of exercise are not yet fully understood, particularly in HIIT, requiring further exploration [153]. Secondly, the dynamic changes of H_2_S during long-term exercise adaptation and its long-term impacts on metabolic health lack systematic research [154]. Moreover, the interactions between the CSE/H_2_S signaling pathway and other gaseous signaling molecules (such as NO and CO) have not been sufficiently elucidated, necessitating in-depth investigation [129].

### 8.2. Technological and Methodological Advances

In recent years, significant advancements have been made in methods for detecting H_2_S levels and activity. Novel fluorescent probes and chromatographic–mass spectrometric techniques (such as GC-MS and LC-MS/MS) have enhanced the sensitivity and specificity of H_2_S detection, enabling real-time monitoring of H_2_S in biological systems [155]. Additionally, nanotechnology-based sensors have been developed for high-throughput and high-precision H_2_S detection, further advancing H_2_S research [156].

Multi-omics approaches have played a crucial role in uncovering new aspects of the CSE/H_2_S signaling pathway. Integrative analyses of genomics, transcriptomics, proteomics, and metabolomics have helped scientists comprehensively understand the complex mechanisms of H_2_S [157]. For example, combining transcriptomic and proteomic data allows for the identification of key genes and proteins regulated by H_2_S, revealing their specific functions in metabolism and signal transduction [158]. Moreover, the application of metabolomics has unveiled the broad impacts of H_2_S on energy metabolism networks, facilitating a deeper understanding of its metabolic regulatory functions [159].

### 8.3. Future Research Directions

Future studies should explore the following key questions:

How does chronic exercise influence H_2_S metabolism?

What is the dose–response relationship for H_2_S supplementation in improving exercise performance and metabolic health?

What biomarkers of H_2_S activity can be used to assess its role in exercise adaptations?

How does H_2_S interact with other gasotransmitters like NO in exercise?

## 9. Conclusions

In conclusion, the CSE/H_2_S signaling pathway plays a crucial role in enhancing muscle function, improving insulin sensitivity, and promoting metabolic health during exercise. By regulating important processes like mitochondrial biogenesis, glucose metabolism, and inflammation, H_2_S helps drive the positive effects of physical activity. However, there are still several challenges in fully understanding how this pathway can be applied clinically. For example, the exact mechanisms by which H_2_S supports exercise adaptations, particularly in different muscle fiber types and various exercise forms, need more research. Additionally, the potential risks of H_2_S supplementation, such as dose-dependent toxicity and interactions with other signaling pathways, must be carefully evaluated.

The therapeutic potential of targeting the CSE/H_2_S pathway is promising, especially in preventing metabolic diseases like type 2 diabetes, improving athletic performance, and aiding muscle recovery after exercise. However, more studies are needed to determine the best dosing strategies, identify reliable biomarkers for H_2_S activity, and assess its long-term effects in clinical settings. Furthermore, exploring how H_2_S interacts with other gasotransmitters, such as NO, could provide valuable insights and create new opportunities for therapeutic development.

Ultimately, advancing our understanding of CSE/H_2_S signaling holds great potential to improve both exercise outcomes and metabolic health. It could lead to personalized exercise programs and targeted therapies for metabolic and muscle-related disorders.

## Figures and Tables

**Table 2:** Key findings from studies on CSE/H_2_S and muscle function.

Research Area	Role of H_2_S	Associated Signaling Pathways and Mechanisms	Potential Impact and Clinical Relevance	References
Regulation of muscle contraction and relaxation	Modulates calcium signaling to influence muscle contraction and relaxation	Activates L-type calcium channels, regulates sarcoplasmic reticulum (RyR, SERCA pumps)	Enhances calcium influx during contraction, improving muscle contraction efficiency and endurance	[81,82,83,84,85,86]
Mitochondrial function and energy metabolism	Stimulates mitochondrial biogenesis and enhances ATP production, reducing ROS generation	Upregulates PGC-1α, NRF1, TFAM; modulates electron transport chain (ETC)	Improves muscle energy production, reduces oxidative damage, and enhances endurance	[48,87,88,89,90]
Muscle repair and regeneration	Promotes satellite cell activation and proliferation for muscle repair and regeneration	Modulates Notch, Wnt, IGF-1/AKT pathways, promoting cell proliferation and differentiation	Accelerates muscle recovery post-exercise, reducing muscle damage	[91,92,93,94,95,96,97]
Anti-apoptotic effects	Inhibits apoptotic pathways to protect muscle cells from damage	Upregulates Bcl-2, inhibits Bax, caspases, and activates PI3K/AKT, ERK1/2 pathways	Maintains muscle cell integrity, reduces exercise-induced cell death	[98,99,100,101,102,103,104,105,106]
Protein synthesis and degradation regulation	Promotes protein synthesis and reduces protein degradation to support muscle growth and strength	Activates mTOR signaling, modulates MyoD and myogenin expression, inhibits UPS and ALP pathways	Enhances muscle hypertrophy, strength, and reduces protein degradation	[107,108,109,110,111,112,113]
Metabolism and protein homeostasis	Regulates FoxO transcription factors to inhibit muscle atrophy	Inhibits FoxO activity, reducing the expression of genes involved in protein degradation	Maintains muscle mass, prevents atrophy, especially after intense physical activity	[114,115,116,117,118]

**Table 3:** Comparison of different H_2_S donors and their effects on exercise performance.

H_2_S Donor	Effects on Exercise Performance	Effects on Metabolic Health	References
NaHS	Increases in vivo H_2_S levels rapidly, suitable for acute studies	Enhances aerobic capacity, increases muscle strength and power, improves recovery	[137]
GYY4137	Improves endurance performance, increases running time and oxygen consumption	Enhances insulin sensitivity, promotes fatty acid oxidation, reduces fat synthesis	[138,140]
AP39	Enhances mitochondrial function and ATP production, improving endurance	Improves glucose metabolism, reduces oxidative stress	[139]
JK1	Increases exercise performance by modulating oxidative stress and muscle function	Promotes muscle fiber hypertrophy, improves metabolic health	[139]
Dietary sources	Supports aerobic performance and muscle strength through increased H_2_S production	Enhances insulin sensitivity, improves lipid profile and glucose metabolism	[141,142,143]
Endogenous H_2_S production	Supports exercise performance by maintaining H_2_S levels through endogenous pathways	Improves metabolic health by supporting glucose uptake and reducing adiposity	[142,143]

## Data Availability

All relevant materials are presented in this manuscript.
